# Dicer protein levels elevated by mild hyperthermia promote a pro-survival phenotype

**DOI:** 10.18632/oncotarget.17433

**Published:** 2017-04-26

**Authors:** Anand S. Devasthanam, Thomas B. Tomasi

**Affiliations:** ^1^ Laboratory of Molecular Medicine, Department of Immunology, Roswell Park Cancer Institute, Buffalo, NY 14263, USA; ^2^ Department of Medicine, State University of New York, School of Medicine and Biomedical Sciences, Buffalo, NY 14214, USA; ^3^ Department of Microbiology and Immunology, State University of New York, School of Medicine and Biomedical Sciences, Buffalo, NY 14214, USA

**Keywords:** hyperthermia, dicer, ER stress, eIF2α, thermotolerance

## Abstract

Cellular exposure to mild stress (39.5°C - 41.5°C) induces thermotolerance, rendering cells resistant to a subsequent heat shock (>42°C) insult. We found that mild hyperthermia at 39.5°C leads to elevations in dicer, a protein well-known for its role in microRNA processing and for its role in cellular stress responses. However, whether elevated dicer protein levels play a role in sustaining a thermotolerant phenotype has, to our knowledge, not been reported. Here we demonstrate that elevated dicer protein is linked to a thermotolerant phenotype in the cervical carcinoma cell line HeLa and in murine embryonic fibroblasts (MEF), and demonstrate that dicer plays a role in mediating PKR and eIF2α phosphorylation. These findings suggest that dicer's role in thermotolerance may be to relay signals to key ER stress pathway components. Moreover, utilizing a MEF cell line defective in microRNA processing, we suggest that dicer's influence on PKR and eIF2α phosphorylation is likely distinct from its microRNA processing role. ATF4 and CHOP are well characterized stress response factors proximal to eIF2α. Evidence is presented that elevated dicer protein in thermotolerant cells differentially modulates ATF4 and CHOP levels to promote a pro-survival phenotype. This work contributes new information on dicer's role in cellular stress responses by defining a pro-survival phenotype in heat stress resistant cells which is sustained, at least in part, by elevated dicer protein levels. Our results suggest an ancillary role for dicer in the cellular stress pathways activated by mild hyperthermia that is likely distinct from its role in microRNA processing.

## INTRODUCTION

Mammalian cells respond to hyperthermia by activating adaptive mechanisms designed to preserve cell viability. In response to mild hyperthermic stress, the Heat Shock Response (HSR) and the endoplasmic reticulum (ER) stress response pathways are activated and preserve the integrity of important signaling networks to sustain cell survival under stressful conditions [1–3]. Despite these finely-orchestrated pro-survival mechanisms, cell death ensues as a consequence of persistent cell stress. For instance, hyperthermic stress in the heat shock range (>42°C) leads to protein denaturation, cell cycle arrest, inhibition of protein and DNA synthesis, as well as disruption of the membrane cytoskeleton with uncoupling of oxidative phosphorylation [4]. In contrast exposing cells to mild hyperthermia (39.5°C - 41.5°C) for extended periods (3-24h) may confer thermotolerance, a transient state of resistance to subsequent hyperthermia treatments [5–8].

The thermotolerant phenotype results from the cumulative outcome of multiple cytoprotective events which occur within a mammalian cell when thermo-tolerance is induced experimentally [9–11]. Recent studies suggest that mild hyperthermia at 40°C for 3-6h leads to ER pathway activation [3, 12]. The ER membrane-resident serine/threonine kinase, PKR-like ER-regulated kinase (PERK), inositol requiring protein 1α (IRE1α) and activating transcription factor (ATF6) are three stress sensors located in the ER membrane [13]. The IRE1α and ATF6 arms work synergistically to transactivate ER chaperone genes, including Grp94, Erp72, BiP and CypB, among others [14]. The ER chaperones function cooperatively to sustain the integrity of important signal transduction events, and increase the folding capacity of the ER. PERK phosphorylates the alpha subunit of the eukaryotic translation initiation factor 2α (eIF2α) resulting in the global suppression of protein synthesis [15]. Although not fully characterized, the protein kinase R (PKR) - a close relative of PERK [16] - is also known to phosphorylate eIF2α during ER stress.

An important consequence of eIF2α phosphorylation is the selective translation of the pro-survival factor, activating transcription factor 4 (ATF4) [17]. ATF4 is a basic leucine zipper (bZIP) protein that can form heterodimers with other bZIP transcription factors, including FOS, JUN and the C/EBP homologous protein (CHOP) [18]. In response to elevated levels of cellular stress, the CHOP protein is stabilized, leading to the elimination of stressed cells via apoptosis [13]. The complete activation of CHOP correlates with the cleavage of ER-associated caspases 12 and 4; phosphorylation of pro-apoptotic kinases ASK1 and JNK; inactivation of the pro-survival factors Bcl-2 and Bcl-xL; and the activation of the mitochondrial apoptotic pathway [19–22]. These events collectively lead to the cleavage of the terminal caspases, including caspase-3.

Cellular stress responses also reveal a role for dicer. The following studies suggest that stress-induced changes in dicer protein levels may influence stress response pathways, leading to cellular resilience under stressful conditions: Ho et al. demonstrated that the decrease in dicer protein levels observed following hypoxia treatments is causally linked to an increase in HIF-1 [23]; Asada et al. showed that a decrease in dicer protein as observed with serum deprivation - a well-known ER stress inducer - correlates with poor cell survival. Interestingly, this study also showed that the ectopic overexpression of dicer augments cell survival under low serum conditions [24].

In previous work, our group showed [25] that mild heat stress for up to 9h at 39.5°C induced increases in the protein levels of dicer in human (BT-20, HeLa, JAR, MRC-5, RAJI, SW-13) and murine (B16, SM9) cell lines. We also observed elevated dicer protein levels in primary cultures derived from mouse renal epithelial cells and mouse renal fibroblasts. Whether mild (39.5°C) hyperthermia treatments also induce thermotolerance, in which dicer plays a role, is currently unclear.

The above observations prompted us to ask: does dicer have a role in influencing stress response pathways during experimentally induced thermotolerance? We hypothesized that the thermotolerant phenotype is sustained, in part, by elevated dicer protein levels. Here we show that mild (39.5°C) hyperthermia-induced increases in dicer protein levels in HeLa and MEF cells are associated with a thermotolerant phenotype. Also reported here is that the increases in dicer protein levels are linked to eIF2α phosphorylation in thermotolerant cells. Moreover, we demonstrate that the role of dicer in mediating eIF2α phosphorylation is likely distinct from its role in microRNA processing. Our results also suggest that the increases in dicer protein levels may play a role in sustaining enhanced ATF4, while curbing CHOP protein levels in thermotolerant cells heading toward heat shock-induced cell death. Collectively, our results support the hypothesis that mild (39.5°C) hyperthermia-induced increases in dicer protein levels in thermotolerant cells may be driving a pro-survival program, thus favoring a cytoprotective outcome.

## RESULTS

### Elevated dicer protein levels observed during mild (39.5°C) hyperthermia stress are linked to a thermotolerant phenotype in HeLa and murine embryonic fibroblast (MEF) cell lines

Prior work showed that 6h heating at 39.5°C increased dicer protein levels in the epithelial cell line HeLa, and in fibroblast cells of human and murine origin [25]. Whether the increase in dicer protein levels observed at 6h of heating at 39.5°C is associated with a thermotolerant phenotype is currently unclear. In addition, whether dicer protein levels remain elevated with extended periods of heating at 39.5°C has not, to our knowledge, been determined.

**Figure 1:**
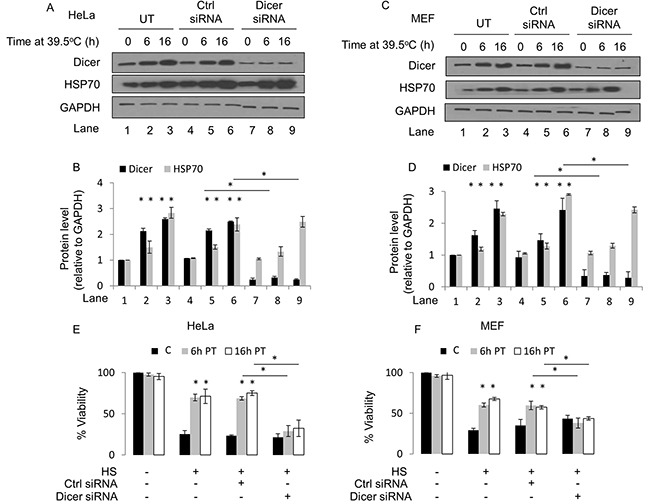
Elevated dicer protein levels are linked to a thermotolerant phenotype in HeLa and murine embryonic fibroblast (MEF) cell lines **(A)** Western blot analysis of dicer, HSP70 and GAPDH protein levels in HeLa cells at 39.5°C for 0h, 6h and 16h. Experiments were conducted under untransfected (UT), control-transfected (Ctrl siRNA) or dicer knockdown (dicer siRNA) conditions. **(B)** Protein band intensities in (A) were first normalized to the GAPDH band intensities corresponding to the specific protein and further normalized to the 0h time point in the untransfected group within each biological replicate. **(C)** Western blot analysis of experiments in MEF cells conducted as described in **(A)**. **(D)** Band intensities in **(C)** analyzed as described in **(B)**. **(E)** MTT cell viability assays in control (37°C) HeLa cells heated at 43°C **(C)**; mild hyperthermia (6h at 39.5°C) pretreated (PT) cells subsequently heated at 43°C (6h PT); and mild hyperthermia (16h at 39.5°C) pretreated cells subsequently heated at 43°C (16h PT). **(F)** MTT cell viability assays in MEF cells conducted as described in **(E)**. The representative western blot images for GAPDH correspond to the western blot image for dicer in this figure. Values shown are mean ± SEM. n = 3, *p < 0.05.

Bettaieb and colleagues have shown that heating HeLa cells for 3h or 6h at 40°C induces a thermotolerant phenotype [7]. This prompted us to ask: are HeLa and MEF cells pretreated at 39.5°C for 6h better able to survive a heat shock (43°C) treatment delivered over a period of 2h? In addition, to determine the effect of more extended periods of heating on dicer protein levels we incubated cells at 39.5°C for 16h. Our results showed that heating HeLa cells for 6h and 16h leads to 2.1-fold and 2.5-fold increase in dicer protein levels when averaged over three independent experiments (Figure 1A and 1B, lanes 1-3). Under identical experimental conditions, the fold changes in dicer protein levels, in MEFs were 1.6-fold and 2.4-fold respectively (Figure 1C and 1D, lanes 1-3).

**Figure 2:**
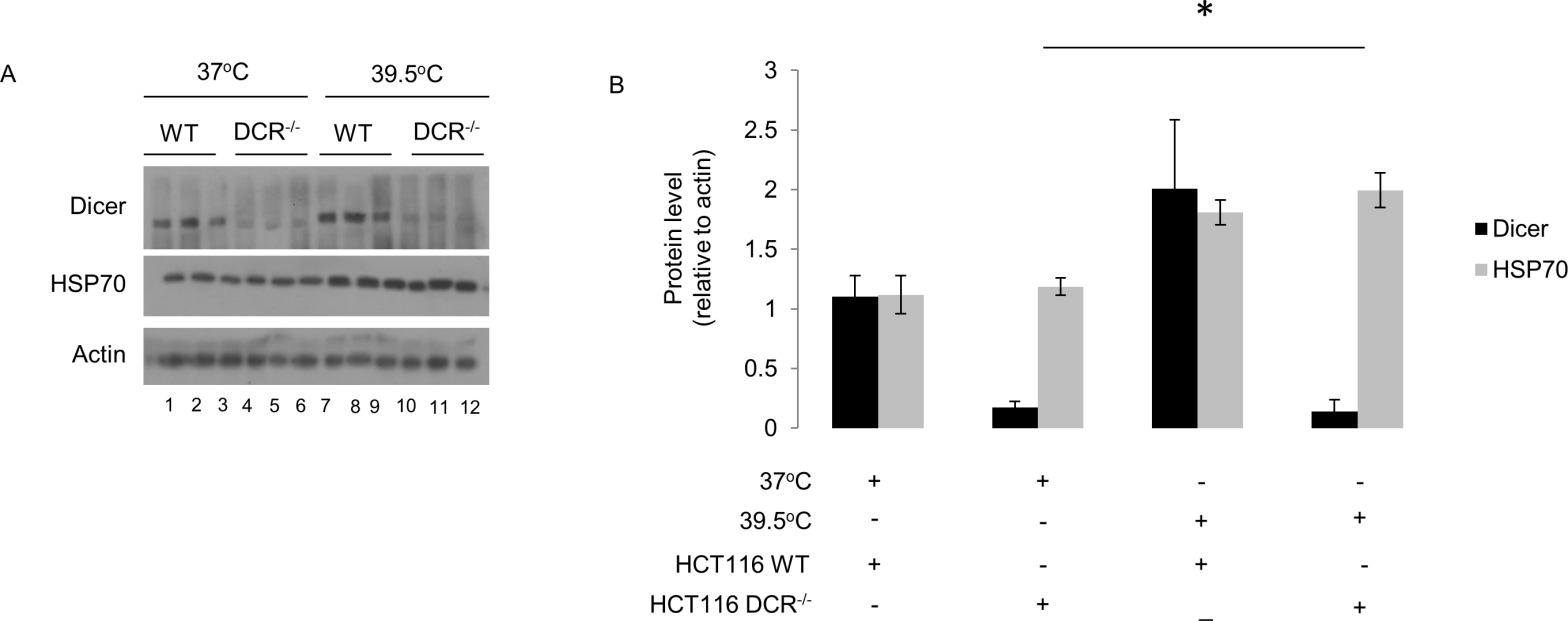
Elevated dicer protein levels observed during mild (39.5°C) hyperthermia-induced thermotolerance are not linked to HSP70 protein levels **(A)** Western blot analysis of HSP70 protein levels in wildtype (WT) and dicer deficient (DCR−/−) HCT116 cells at mild hyperthermia (39.5°C) for 16h. **(B)** Protein band intensities in **(A)** were first normalized to the actin band intensities corresponding to the specific protein and further normalized to the first experimental replicate in control (lane 1) and mild hyperthermia (lane 7) samples within each biological replicate. The representative western blot images for Actin correspond to the western blot image for dicer in this figure. Values shown are mean ± SEM. p < 0.05.

To gain insight into the biological relevance of elevated dicer protein levels observed in cells treated with mild hyperthermia at 39.5°C, we conducted knockdown studies in HeLa and MEF cell lines. We first verified that dicer knockdown via siRNA leads to a significant reduction in the steady state levels of the dicer protein in HeLa cells without resulting in cell death. We heated control-transfected as well as dicer knockdown HeLa and MEF cells at 39.5°C for 6h and 16h and then challenged the heated cells with a cytotoxic heat shock treatment. To ensure that heat shock treatments induced cell death, a positive control group that received heat shock treatments alone was included. We conducted MTT cell viability assays and determined the proportion of viable cells under each treatment condition. Our results suggest that HeLa and MEF cells treated under mild hyperthermia at 39.5°C for 6h or 16h become thermotolerant, as evidenced by a significant increase in the proportion of cells surviving a heat shock challenge (Figure 1E and 1F). These results are consistent with previous reports [7] suggesting that treating cells with mild hyperthermia stress is a method of inducing thermotolerance in an experimental setting. The results from the dicer knockdown studies suggest that the elevations in dicer protein levels are linked to a thermotolerant phenotype, shown by the significant decrease in the proportion of dicer knockdown HeLa and MEF cells surviving a heat shock challenge (Figure 1E and 1F). It is important to note that dicer knockdown in HeLa and MEF cells heated at 39.5°C for 6h and 16h did not significantly alter the induction of HSP70 protein levels. When stabilized by thermal stress, HSP70 plays an important role in conferring a thermotolerant phenotype [28]. As an initial step in determining the mechanism by which dicer may potentially stabilize HSP70 protein levels during hyperthermia stress, we asked whether dicer is required for hyperthermia-induced increases in HSP70 protein levels. To this end, we heated wildtype (WT) and dicer deficient (DCR^−/−^) HCT116 cells at mild hyperthermia for 16h and found that HSP70 protein levels increased in WT as well as in dicer deficient cell lines (Figure 2). These results suggest that the role of dicer in mild hyperthermia-induced thermotolerance likely does not involve the modulation of HSP70 protein levels.

### Elevated dicer protein levels observed during mild (39.5°C) hyperthermia-induced thermotolerance are linked to PKR and eIF2α phosphorylation in HeLa cells

To gain further insight into the role of dicer in thermotolerance, we performed studies to address the potential role of dicer in pathways relevant to the ER stress response. Previous studies showing that mild hyperthermia at 40°C is associated with the phosphorylation of eukaryotic Initiation Factor 2 alpha-subunit (eIF2α) on Serine 51 [3, 12] prompted us to ask: are elevated dicer protein levels causally linked to the activation of key components in the ER stress pathway?

**Figure 3:**
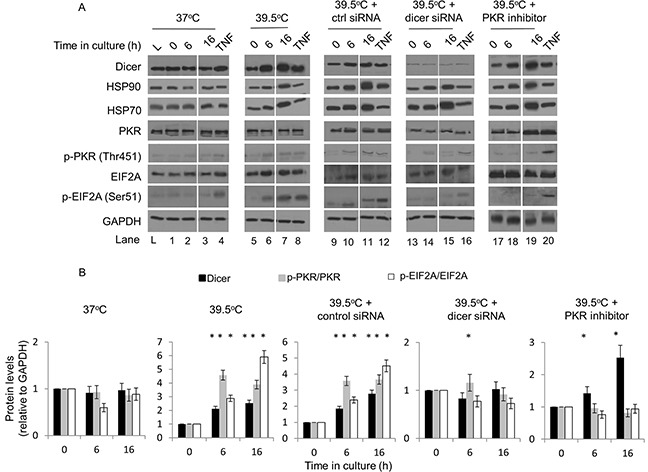
Elevated dicer protein levels observed during mild (39.5°C) hyperthermia-induced thermotolerance are linked to PKR and eIF2α phosphorylation in HeLa cells **(A)** Western blot analysis of dicer, phosphorylated and total PKR and eIF2α at 37°C and at 39.5°C in the presence of control or dicer siRNA. Mild hyperthermia treatments were initiated 48h post-transfection. TNF-alpha (10 ng/ml for 2h) was used as the positive control for phosphorylated PKR and eIF2α. A PKR inhibitor (250 nM) was added 1h before mild hyperthermia treatments were initiated. The lane ‘L’ indicates a commercial source of HeLa cell lysate that was used as a comparator to confirm that cells used in our experiments did not have detectable levels of active PKR in the absence of cell stress. PKR inhibitor was added to samples heated for 0h, 6h and 16h (i.e. lanes 17, 18 and 19). **(B)** Protein band intensities in **(A)** were first normalized to the GAPDH band intensities corresponding to that specific protein and further normalized to the 0h time point in the respective experimental group. Breaks in western blot images were created to juxtapose time points pertinent to the experimental question. The ratios of phosphorylated to total proteins are graphed. The representative western blot images for GAPDH correspond to the western blot image for dicer this figure. Values shown are mean ± SEM. n = 3, *p < 0.05.

Under control (37°C) conditions, there were no detectable changes in phosphorylated PKR (Thr451) and eIF2α (Ser51) (Figure 3A, lanes 1-3, and Figure 3B). As a positive control, we utilized TNF-α which is known to induce phosphorylation of eIF2α via PKR [26] (Figure 3A, lane 4, and Figure 3B). Additionally, at 39.5°C we observed significant increases in phosphorylated PKR as well as eIF2α at 6h and 16h time points (Figure 3A, lanes 5-7, and Figure 3B). Interestingly, the increases observed in dicer protein levels appeared to coincide with the phosphorylation status of PKR as well as eIF2α. This observation was consistent in cells transfected with a control siRNA (Figure 3A, lanes 9-11, and Figure 3B). Potentially important is the observation that dicer knockdown led to a significant reduction in the phosphorylation of eIF2α as well as PKR in cells heated at 39.5°C for 6h and 16h (Figure 3A, lanes 13-15, and Figure 3B).

EIF2α can be phosphorylated by four kinases: PKR, HRI, PEK and GCN2 [27]. This prompted us to ask: does experimentally induced thermotolerance lead to the activation of eIF2α via a PKR-dependent mechanism; and are the observed increases in dicer protein levels causally linked to the phosphorylation of eIF2α? To address this question, we analyzed the protein levels of dicer as well as phosphorylated PKR and eIF2α in cells at 39.5°C that were pretreated with a small molecule inhibitor of PKR. Our results showed that PKR inhibition significantly suppressed the phosphorylation of PKR and eIF2α despite the increases in dicer protein levels (Figure 3A, lanes 18-19, and Figure 3B). We also verified that the PKR inhibitor alone (250nM for 16h) under control (37°C) conditions did not significantly affect dicer protein levels (data not shown).

Taken together, these results suggest that PKR and eIF2α phosphorylation are observed during experimentally induced thermotolerance in HeLa cells, and that PKR may be mediating eIF2α phosphorylation in thermotolerant cells. Moreover, our results support the hypothesis that the elevations in dicer protein levels are linked to the phosphorylation of PKR and eIF2α during mild (39.5°C) hyperthermia-induced thermotolerance.

### The role of dicer in regulating PKR and eIF2α phosphorylation during mild (39.5°C) hyperthermia-induced thermotolerance is likely distinct from its role in microRNA processing

To obtain further mechanistic insight into whether experimentally induced thermotolerance influences dicer activity, we conducted a dicer activity assay with HeLa cells treated with mild hyperthermia for 6h and 16h.

Dicer cleaves pre-microRNAs (∼70nt) via its RNase III domains to form mature microRNAs (∼22nt). To determine whether the elevations in dicer protein levels observed during experimentally induced thermotolerance correlate with dicer activity, we conducted an *in vitro* dicer activity assay designed to measure the cleavage of a radiolabelled pre-microRNA substrate (88nt) to a mature microRNA product (22nt). Pre-miR-124a was chosen as the substrate for this assay because it is not generated endogenously in the cell types used in this study.

**Figure 4:**
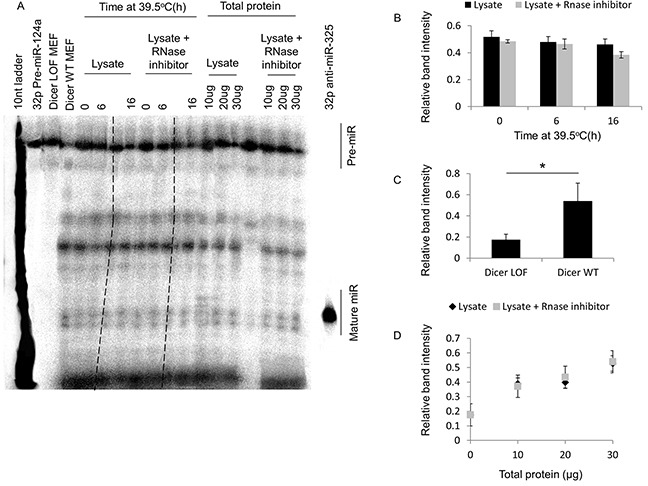
Mild hyperthermia (39.5°C) stress-induced thermotolerance does not significantly alter dicer activity in HeLa cells **(A)** Urea PAGE analysis of radiolabeled pre-microRNA-124a and mature microRNA-124a in HeLa cells at 39.5°C for 0h, 6h and 16h. Gel lanes excluded from analysis are crossed out with broken lines. **(B)** The ratio of the band intensities of the mature microRNA to that of the pre-microRNA (i.e. relative 22nt mature MicroRNA band intensity) in lanes containing cell lysate alone, or cell lysate supplemented with RNase inhibitor. **(C)** Relative band intensities of lanes containing lysates from dicer LOF and wildtype (WT) MEFs. **(D)** Relative band intensities of lanes containing 10, 20 and 30 ug of total protein from lysate alone, or lysate supplemented with RNase inhibitor. Lanes excluded from the analysis are marked with broken lines. Values shown are mean ± SEM. n = 3; *p < 0.05.

Our results from the dicer activity assay suggest that there are no significant differences in the band intensities corresponding to the 22nt product in samples from the 6h and 16h heating groups (Figure 4A and 4B). To ensure that the activity assay was specific to dicer, we analyzed the differences in the band intensities corresponding to the mature microRNA product between whole cell lysates derived from wildtype (WT) and dicer LOF MEFs (Figure 4A and 4C). The expression of dicer was confirmed in both WT and dicer LOF MEF lysates (data not shown). Reactions contained 20 micrograms of total protein derived from mild (39.5°C) hyperthermia treated HeLa cells. Whole cell protein lysates containing 10, 20 and 30 micrograms of total protein derived from HeLa cells were included to show that the amount of protein in the reaction did not saturate the assay (Figure 4A and 4D). Taken together, we conclude that the elevations in dicer protein levels observed during experimentally induced thermotolerance do not lead to enhanced dicer activity.

The data also support the idea that increases in dicer protein levels alone are sufficient to mediate PKR and eIF2α phosphorylation in thermotolerant cells. To test this, we asked whether elevated levels of the dicer protein, rather than its ability to process microRNAs, might have a greater impact on mediating PKR and eIF2α phosphorylation in thermotolerant cells. We analyzed the phosphorylation of PKR and eIF2α in WT and in dicer LOF MEF cell lines heated at 39.5°C for 16h. In initial studies, we found that 16h of heating led to greater increases in dicer protein levels compared to 6h of heating in MEF cells (Figure 1). Therefore, we focused on the 16h time point to explore a potential causal link between elevations in dicer protein levels and phosphorylation events on PKR and eIF2α.

**Figure 5:**
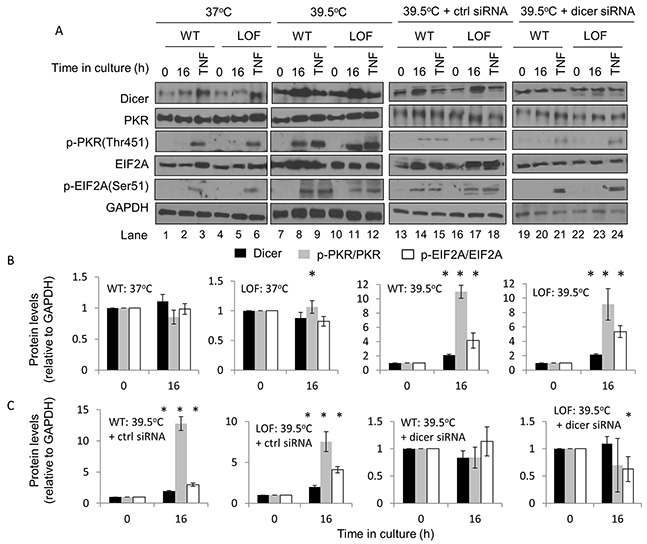
The role of dicer in influencing PKR and eIF2α phosphorylation during mild (39.5°C) hyperthermia-induced thermotolerance is likely distinct from its role in microRNA processing **(A)** Western blot analysis of dicer, total and phosphorylated PKR, total and phosphorylated eIF2α and GAPDH in WT and dicer LOF MEFs at 0h and 16h at 39.5°C under untransfected (lanes 1-12), control-transfected (lanes 13-18), and dicer knockdown (lanes 19-24) conditions. **(B)** Protein band intensities in (**A**: lanes 1-12) were first normalized to GAPDH band intensities corresponding to that specific protein and further normalized to the 0h time points in the respective experimental group. **(C)** Band intensities in (**A**: lanes 13-24) were analyzed by the same method described in **(B)**. The ratios of phosphorylated to total proteins in **(A)** are graphed in **(B)** and **(C)**. The representative western blot images for GAPDH correspond to the western blot image for dicer this figure. Values shown are mean ± SEM. n = 3, *p < 0.05.

Under control (37°C) conditions, there were no significant differences in dicer protein levels between WT and dicer LOF cells (Figure 5A, lanes 1-6, and Figure 5B), and also no significant differences in phosphorylated eIF2α levels between WT and dicer LOF cells. Consistent with our results in HeLa cells, WT MEFs that were heated for 16h at 39.5°C showed significant increases in dicer, phosphorylated PKR and phosphorylated eIF2α. Consistent with results in WT MEFs, dicer LOF MEFs that were heated for 16h at 39.5°C also showed significant increases in dicer, phosphorylated PKR and phosphorylated eIF2α (Figure 5A, lanes 7-12, and Figure 5B). These results suggest that elevated dicer protein observed during experimentally induced thermotolerance is associated with increases in phosphorylated PKR and phosphorylated eIF2α in MEFs expressing functionally defective dicer protein.

To determine whether elevated levels of the dicer protein itself could sustain PKR and eIF2α phosphorylation in thermotolerant cells, dicer protein levels were suppressed in dicer LOF MEFs by transfecting cells with a dicer-targeting siRNA. Dicer protein levels and phosphorylated PKR and eIF2α in cells treated under mild (39.5°C) hyperthermia stress for 16h were then analyzed. Our results suggest that, while PKR and eIF2α are phosphorylated in heated WT and LOF cells transfected with a control siRNA (Figure 5A, lanes 13-18, and Figure 5C), these phosphorylation events were largely abrogated in dicer knockdown cells (Figure 5A, lanes 19-24, and Figure 5C). Taken together, the results support the concept that the elevations in dicer protein levels observed during mild (39.5°C) hyperthermia-induced thermotolerance are sufficient to sustain phosphorylation events on PKR and eIF2α, and influence the PKR and eIF2α phosphorylation in thermotolerant cells via mechanisms that are likely distinct from its role in microRNA processing.

### Elevated dicer protein levels observed during mild (39.5°C) hyperthermia-induced thermotolerance influence ATF4 and CHOP protein levels in HeLa and MEF cells

Stress-induced increases in ATF4 protein levels lead to the activation of ATF3, CHOP, HO-1, RANKL and VEGF – factors that play important roles in stress remediation pathways collectively referred to as the integrated stress response [49]. It is now recognized that elevated ATF4 protein levels are primarily associated with a pro-survival outcome. In contrast, stress-induced increases in CHOP protein levels are associated with a pro-apoptotic outcome, as evidenced by a direct correlation between elevated CHOP protein levels and the activation of GADD53, TRIB3, IL6, PUMA and BIM and terminal caspases, including caspase-3 [50, 51]. Since thermotolerance is thought to be a form of stress adaptation, are the elevations in dicer protein levels influencing ATF4 and CHOP protein levels?

**Figure 6:**
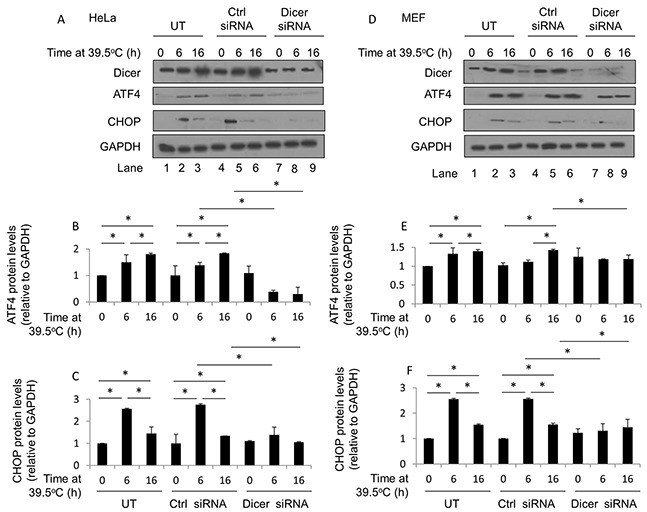
Elevated dicer protein levels observed during mild (39.5°C) hyperthermia-induced thermotolerance influence ATF4 and CHOP protein levels in HeLa and MEF cells **(A)** Western blot analysis of dicer, ATF4, CHOP and GAPDH protein levels in HeLa cells at 39.5°C for 0h, 6h and 16h under untransfected (UT), control-transfected (Ctrl siRNA), and dicer knockdown (dicer siRNA) conditions. **(B)** ATF4 band intensities in **(A)** were first normalized to GAPDH band intensities corresponding to ATF4 (not shown) and further normalized to the 0h value in the untransfected group. **(C)** CHOP band intensities in **(A)** were first normalized to GAPDH band intensities corresponding to CHOP (not shown) and further normalized to the 0h value in the untransfected group. **(D)** Western blot analysis as described in **(A)** conducted in MEF cells **(E)**. ATF4 band intensity analysis as described in **(B)** conducted in MEF cells **(F)**. CHOP band intensity analysis as described in (C) conducted in MEF cells. The representative western blot images for GAPDH correspond to the western blot image for dicer this figure. Values shown are mean ± SEM. n = 3, *p < 0.05.

To address this question, HeLa and MEF control-transfected and dicer knockdown cells were heated for 6h and 16h at 39.5°C and protein levels of ATF4 and CHOP analyzed. We observed increases in ATF4 protein levels in HeLa cells heated at 39.5°C for 6h and 16h (Figure 6A, lanes 1-6, and Figure 6B). An analysis of CHOP protein levels in HeLa cells showed increases in CHOP protein at 6h of heating at 39.5°C, which declined significantly by the 16h time point (Figure 6A, lanes 1-6, and Figure 6C). The increases in ATF4 and CHOP protein levels were largely suppressed in dicer knockdown HeLa cells (Figure 6A, lanes 7-9, and Figure 6B and 6C). Also, as with the observations in HeLa cells, CHOP protein levels at 16h of heating at 39.5°C were significantly lower than those at the 6h time point in MEF cells (Figure 6D, lanes 1-6, and Figure 6F). Importantly, these increases were largely suppressed in dicer knockdown MEF cells (Figure 6D, lanes 7-9, and Figure 6E and 6F). Taken together, our results support the notion that elevations in dicer protein levels observed during mild (39.5°C) hyperthermia-induced thermotolerance influence ATF4 and CHOP protein levels.

### Elevated dicer protein levels observed during mild (39.5°C) hyperthermia-induced thermotolerance is associated with a pro-survival phenotype in HeLa and MEF cells

The question of whether a potential link exists between elevated dicer protein levels and ATF4 and/or CHOP protein levels in thermotolerant cells challenged with persistent cell stress was addressed by carrying out time course experiments in thermotolerant HeLa and MEF cells with varying time points of heat shock at 43°C.

**Figure 7:**
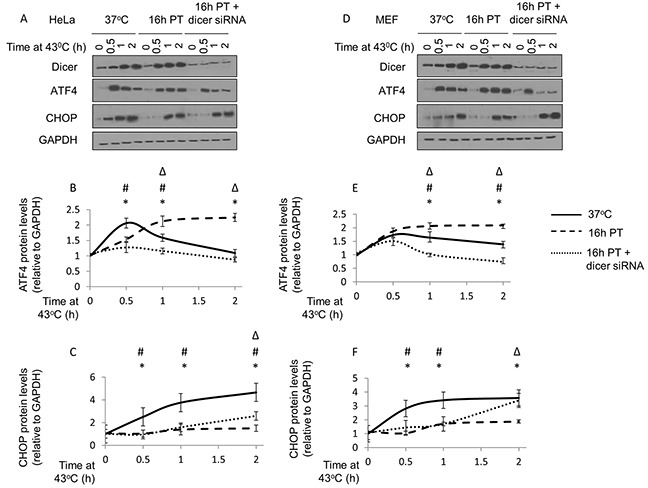
Elevated dicer protein levels observed during mild (39.5°C) hyperthermia-induced thermotolerance is associated with a pro-survival phenotype in HeLa and MEF cells **(A)** Western blot analysis of dicer, ATF4, CHOP and GAPDH protein levels in control (37°C), mild hyperthermia (39.5°C) pretreated (16h PT), and mild hyperthermia pretreated dicer knockdown (16h PT + dicer siRNA) HeLa cells challenged with a heat shock (43°C) insult for 0, 0.5, 1 and 2h. **(B)** ATF4 band intensities in **(A)** were first normalized to GAPDH band intensities corresponding to ATF4 (not shown) and further normalized to the 0h time point in the control (37°C) group. Statistically significant (p<0.05) differences between control and 16hPT (*); control and 16hPT + dicer siRNA (#); and 16hPT and 16h PT + dicer siRNA (Δ) are displayed. **(C)** CHOP band intensities in **(A)** were first normalized to GAPDH band intensities corresponding to CHOP (not shown) and analyzed as described in **(B)**. **(D)** Western blot analysis as described in **(A)** conducted in MEF cells. **(E)** ATF4 band intensity analysis as described in **(B)** conducted in MEF cells. **(F)** CHOP band intensity analysis as described in **(C)** conducted in MEF cells. The representative western blot images for GAPDH correspond to the western blot image for dicer in this figure. Values shown are mean ± SEM. n=3, *p<0.05 (37°C v/s 16hPT), #p<0.05 (37°C v/s 16hPT + dicer siRNA), Δp<0.05 (16hPT v/s 16hPT + dicer siRNA).

The results suggest that experimentally induced thermotolerance (i.e. 39.5°C, 16h) restructures ATF4 and CHOP induction patterns in cells subsequently challenged with a heat shock insult. Specifically, heat shock results in increased ATF4 (Figure 7A and 7B, compare solid and dotted lines) and suppressed CHOP (Figure 7A and 7C, compare solid and dotted lines) in thermotolerant HeLa cells. While there were differences in the patterns of induction in MEF cells of ATF4 (Figure 7D and 7E) and CHOP (Figure 7D and 7F), results in MEF cells were consistent with those observed in HeLa cells.

Our data also suggest that in HeLa cells under thermotolerance-inducing conditions (i.e. 39.5°C, 16h), dicer knockdown appeared to constrain increases in ATF4 protein levels (Figure 7A and 7B, compare broken and dotted lines). Under similar experimental conditions, dicer knockdown was associated with increases in CHOP protein levels (Figure 7A and 7C, compare broken and dotted lines). Results in MEF cells were consistent with those observed in HeLa cells (Figure 7D-7F).

Collectively, these results suggest that the pro-survival phenotype (i.e. increased ATF4, suppressed CHOP) associated with experimentally induced thermotolerance is reversed (i.e. decreased ATF4, increased CHOP) in dicer knockdown cells.

Since dicer knockdown diminished the increases in the pro-survival factor ATF4, and coincided with increases in the pro-apoptotic factor CHOP in thermotolerant cells challenged with a heat shock insult, we believe that the elevated dicer protein levels observed during mild (39.5°C) hyperthermia-induced thermotolerance play a role in establishing a pro-survival phenotype. Therefore, we reason that the elevated dicer protein levels observed during mild (39.5°C) hyperthermia-induced thermotolerance play a role in favoring a pro-survival phenotype by differentially modulating ATF4 and CHOP protein levels.

### Elevated dicer protein levels observed during mild (39.5°C) hyperthermia-induced thermotolerance is associated with a pro-survival outcome in HeLa and MEF cells

A previous study in HeLa cells showing that heating HeLa cells at 40°C confers resistance to heat shock-induced apoptosis [8] prompted the question: do the elevated dicer protein levels, observed during mild (39.5°C) hyperthermia-induced thermotolerance, influence caspase-3 activation in cells challenged with a cytotoxic heat shock insult at 43°C?

**Figure 8:**
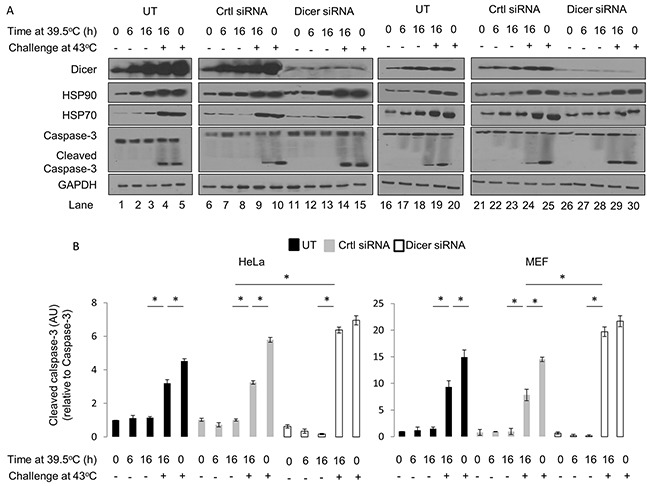
Elevated dicer protein levels observed during mild hyperthermia (39.5°C) induced thermotolerance is associated with a pro-survival outcome in HeLa and MEF cells **(A)** Western blot analysis of dicer, HSP90, HSP70, caspase-3 and cleaved caspase-3 protein levels in HeLa cells pretreated at 39.5°C for 0h, 6h and 16h and subsequently challenged with a heat shock insult at 43°C. Experiments were conducted under untransfected (UT: lanes 1-5), control-transfected (Ctrl siRNA: lanes 6-10), and dicer knockdown (dicer siRNA: lanes 11-15). Identical experiments were conducted in MEF cells (lanes 16-30). **(B)** Caspase-3 and cleaved caspase-3 band intensities in **(A)**, lanes 1-15, were first normalized to GAPDH corresponding to Caspase-3 (not shown) and further normalized to the 0h time point in the untransfected group. The ratios of cleaved caspase-3 to total caspase-3 levels are graphed. Caspase-3 and cleaved caspase-3 band intensity analysis as described in **(B)** conducted with results in MEF cells shown in **(A)**, lanes 16-30. The representative western blot images for GAPDH correspond to the western blot image for dicer this figure. Values shown are mean ± SEM. n=3, *p<0.05.

Mild (39.5°C) hyperthermia alone, or in combination with control or dicer-targeting siRNAs, was not cytotoxic to HeLa cells, as evidenced by the lack of cleaved caspase-3 in these experiments (Figure 8A, lanes 1-3; 6-8, and 12-14). Similar results were observed in MEF cells (Figure 8A, lanes 16-18; 21-23, and 26-28).

Thermotolerant HeLa and MEF cells challenged with a heat shock insult showed consistently lower levels of cleaved caspase-3 compared to cells that received the heat shock treatment alone (Figure 8A, lanes 4-5; 9-10; 19-20; 24-25, and Figure 8B). These results are consistent with our initial observations suggesting that thermotolerant HeLa and MEF cells are better able to withstand a heat shock insult (see Figure 1E and 1F). Importantly, dicer knockdown in HeLa and MEF cells that were heated for 16h at 39.5°C had significantly greater levels of cleaved caspase-3 when challenged with a heat shock insult (Figure 8A, lanes 14-15; 29-30, and Figure 8B). Taken together, our data suggests that preventing mild (39.5°C) hyperthermia-induced increases in dicer protein levels results in an increase in heat shock-induced cleaved caspase-3. This again supports the thesis that elevated dicer protein levels observed during mild (39.5°C) hyperthermia-induced thermotolerance are associated with a pro-survival outcome.

## DISCUSSION

The nexus of this study is the association of mild hyperthermia-induced increases in dicer protein levels with a thermotolerant phenotype and a description of a potential mechanism by which dicer may play a cytoprotective role. We show a link between increased dicer protein levels and the phosphorylation of Protein Kinase R (PKR) and eukaryotic Initiation Factor 2α (eIF2 α), as well as the transactivation and differential modulation of ATF4 and CHOP in favor of a pro-survival phenotype in thermotolerant cells. Furthermore, our work showing an inverse relationship between dicer and cleaved caspase-3 protein levels suggests that dicer may influence both ER and mitochondrial apoptotic pathways. Our results support the notion that the role of elevated dicer protein levels in mediating phosphorylation events might be unrelated to its role in microRNA processing, thereby uncovering a potentially novel mechanism by which dicer protein levels may engage the ER stress network in thermotolerant cells.

It is important to note that dicer knockdown in HeLa and MEF cells heated at 39.5°C for 6h and 16h did not significantly alter the induction of HSP70 protein levels. HSP70 is well known for its role in conferring a thermotolerant phenotype [28]. Gupta et al. demonstrated using chemical inducers of ER stress that HSP72, the inducible isoform of HSP70, synergizes with the IRE1α arm of the ER stress pathway, resulting in a cytoprotective outcome [29]. Whether HSP70 is involved in the ER stress pathway in our experiments is currently unclear and warrants further investigation. A detailed study focusing on the potential role of dicer in the HSR pathway is yet to be reported. Our results showing increases in dicer as well as HSP70 protein levels in HeLa cells (Figure 1A and 1B; lanes 1-6) and MEF (Figure 1C and 1D; lanes 1-6) prompts the question whether dicer plays a role in increasing HSP70 levels. Our knockdown studies in HeLa (Figure 1A and 1B; lanes 7-9) and MEF (Figure 1C and 1D; lanes 7-9) cells suggest that increases in dicer protein levels are not likely a prerequisite for increases in HSP70 levels during mild hyperthermia stress. However, given that dicer knockdown cells still contain dicer, it was unclear whether dicer may be involved in regulating HSP70. As an initial step in determining the mechanism by which dicer may potentially stabilize HSP70 protein levels during hyperthermia stress, we asked whether dicer is required for hyperthermia-induced increases in HSP70 protein levels. To this end, we heated wildtype (WT) and dicer deficient (DCR^−/−^) HCT116 cells at mild hyperthermia for 16h and found that HSP70 protein levels increase in WT and DCR^−/−^ cells (Figure 2), suggesting that HSP70 protein synthesis and regulation may occur independently of changes in dicer protein levels. While our study was limited to analyzing HSP70 protein levels, other studies show that hyperthermia stress in the heat shock range induces several HSP family members, namely HSPs 27, 32, 60, 72 and 110 [30–32]. The patterns of HSP induction during extended periods of mild hyperthermic stress, and the potential role of dicer in influencing HSPs is outside the purview of this report. Future studies aimed at investigating dicer's influence on the HSR could contribute to the establishment of a comprehensive working model for dicer in thermotolerant cells.

Interestingly, our results from PKR inhibitor studies suggest that PKR may play an intermediary role between dicer and eIF2α. Cawley et al., report that eIF2α phosphorylation remains unaltered in dicer knockdown HCT116 cells treated with ER stress-inducing chemicals [33], suggesting that the ER stress pathway can be activated chemically via dicer-independent mechanisms. Our results however, suggest a causal link between elevated dicer protein levels and eIF2α phosphorylation, potentially mediated by PKR. Hence, a question arises as to how dicer protein levels and the PKR-eIF2α axis might be mechanistically linked in the context of mild hyperthermia stress. Although our data does not elucidate a definitive mechanism, we propose that the Protein Activator of PKR (PACT) – a dicer-interacting protein and a strong inducer of PKR activation [34], may be involved in the pathway linking elevated dicer protein levels and the PKR-eIF2α axis during mild (39.5°C) hyperthermia stress. Future work aimed at analyzing the effect of mild hyperthermia stress on the phosphorylation of various PACT serine residues (S18, S246, S287) and their biological outcomes will add to our understanding of the role of dicer in the ER stress pathway during experimentally induced thermotolerance.

Signal transduction is regulated, at least in part, via microRNAs. For example, a study by Sahasrabuddhe et al. shows that the genetic ablation of dicer leads to widespread deregulation in multiple pathways that are strongly dependent on phosphorylation events [35]. Other studies have shown that the eIF2α kinase PKR is subject to regulation by miR-29b, and potentially other microRNAs [36, 37]. The conclusions drawn from our dicer LOF cell line studies are currently limited to one cell line. Future work utilizing genetic ablation approaches will likely clarify the role of dicer in thermotolerance. A study in fission yeast shows that DCR1 (a homologue of human DICER1) acts cooperatively with HSP104 to mitigate thermal stress-induced prion aggregation [38]. It is plausible that the role of human dicer in conferring cellular robustness during thermal stress could represent an evolutionary remnant from an era when thermal stress adaptation was closely linked to survival in harsh environments.

While our studies focused only on ATF4, there are additional factors that regulate CHOP. These factors include ATF2 during leucine starvation; ATF3 during arsenite-induced stress and hypoxia; and ATF6 and XBP-1 in cells exposed to chemical inducers of ER stress [39–41]. A leading study in the field proposes that all of the aforementioned transcription factors are required to achieve maximal induction of CHOP [42]. However, a comprehensive description of the various mechanisms through which CHOP is transcriptionally regulated during mild hyperthermia stress is currently lacking. Here we suggest that elevated dicer protein levels observed during mild (39.5°C) hyperthermia-induced thermotolerance might be influencing the eIF2α-ATF4-CHOP axis and that future studies should include an analysis of the role of dicer in potentially regulating the other known transcriptional regulators of CHOP in thermotolerant cells.

Drawing a clear link between the protein levels of dicer and CHOP is limited by a technical incongruence: bands on the western blot in Figure 7 do not always correspond to the bar graphs in this instance. To address this issue, we have conducted experiments with technical replicates of samples used in experiments pertaining to Figure 7D and have analyzed CHOP protein levels in control (37°C) and in dicer knockdown (16h pre-treated at 39.5°C) MEF cells exposed to heat shock at 43°C for 1h (data not shown). Results from this confirmatory experiment suggest that while there were variations in CHOP protein levels between replicates, the overall outcome was largely consistent with the results shown in Figure 7F: CHOP protein levels in control (37°C) cells exceed those in dicer knockdown (siRNA to dicer + 16h pre-treated at 39.5°C) cells under heat shock (43°C) for 1h.

Although this work implicates a link between mild (39.5°C) hyperthermia-induced increases in dicer protein levels and eIF2α phosphorylation, a potential mechanistic limitation is that a link between dicer protein levels and ATF4 and CHOP remains unclear. To the best of our knowledge, there are no studies in HeLa and MEF cells suggesting that ATF4 can be induced independently of eIF2α phosphorylation. Moreover, the robust nature of the eIF2α-ATF4-CHOP axis is exemplified by the observation that ATF4 and CHOP induction is abrogated in eIF2α S51A cells [41]. On the basis of our results, we propose that dicer is upstream of the eIF2α-ATF4-CHOP axis. If this is the case, a question arises as to whether the ectopic overexpression of dicer would lead to the spontaneous induction of ATF4 and CHOP. To address this question, we overexpressed dicer in HeLa cells and found that neither ATF4 nor CHOP were induced (data not shown). Given that plasmid gene expression is dependent on active protein translation; and given that ATF4 and CHOP are induced upon translation inhibition, it is challenging to demonstrate a direct link between elevated dicer protein levels and ATF4 and CHOP. This work provides a rationale for future studies aimed at determining whether dicer may act as a signaling mediator within the ER stress network.

A study in MEFs using non-cytotoxic doses of the ER stress-inducing chemical tunicamycin showed that CHOP protein levels increase up to 8-fold at 16h after treatment initiation and gradually decline to near-baseline levels by 24h [43]. The Rutkowski study (43) proposes that CHOP non-responsiveness, or the lack of an increase in CHOP protein levels over time, leads to stress adaptation since the cellular abundance of CHOP is insufficient to trigger pro-apoptotic programs. Our results showing an initial increase (6h of heating) and a subsequent decline in CHOP protein levels during an extended period of heating (16h) supports the idea that cells might be adapting to ER stress during experimentally induced thermotolerance. In addition, the lack of an increase in ATF4 and CHOP protein levels in dicer knockdown HeLa and MEF cells supports the concept that elevated dicer protein levels might be playing a role in facilitating cellular stress adaptation mechanisms, potentially by modulating ATF4 and CHOP protein levels.

Dicer protein levels increase with heat shock (Figure 8A; lanes 4, 5, 9, 10, 19, 20, 24 and 25). While a cytoprotective role for dicer has been suggested [23, 24, this report], one study also highlights a role for dicer in programmed cell death downstream of caspase-3 [45]. An important observation in this study was that elevated dicer protein levels observed during mild (39.5°C) hyperthermia stress were associated with a pro-survival outcome. A limitation is that this cannot be extrapolated to interpret that the increases in dicer protein levels observed during the heat shock will continue to sustain pro-survival pathways. The role of dicer in heat shock-induced apoptosis is currently unknown and requires further exploration.

In conclusion, this work provides data that the thermotolerant phenotype is sustained, at least in part, by elevated dicer protein levels observed during mild (39.5°C) hyperthermia-induced thermotolerance. Under these conditions, the elevated dicer protein level is linked to the phosphorylation of eIF2α and the modulation of ATF4 and CHOP to favor a pro-survival outcome. Our study proposes a novel role for dicer in the cellular stress response to mild hyperthermia that is likely distinct from its role in microRNA processing.

## MATERIALS AND METHODS

### Cell lines and culture conditions

HeLa cells (CCL-2) were purchased from the American Type Culture Collection (ATCC) (Manassas, VA) and cultured according to ATCC's instructions. Wildtype and dicer loss-of-function (LOF) MEF cells were a generous gift from Dr. Gaspare La Rocca (Memorial Sloan Kettering) and were cultured in DMEM with 10% FBS, supplemented with 1% L-Glutamine. Both HeLa and murine embryonic fibroblast (MEF) cell culture media were supplemented with an antibiotic/antimycotic cocktail (ThermoFisher Scientific, Grand Island, NY). The DCR^−/−^ cells used in our studies were used as previously described [46]. Our results showing very low levels of a band corresponding to dicer are consistent with previous work by others [47, 48]. WT and DCR^−/−^ HCT116 cells were grown in DMEM supplemented with 10% FBS, as described previously [47].

### Hyperthermia treatments

For mild hyperthermia treatments, cell cultures were incubated in water-jacketed incubators (7% CO2 for HeLa; 5% for MEF) at 39.5°C for the indicated times. For heat shock treatments, cell cultures were incubated in temperature-controlled water baths at 43°C for the indicated times. Heat shocked cells were transferred to water-jacketed incubators at 37°C for 6h following heat shock in experiments that involved the visualization of cleaved caspase-3. A study on heat shock-induced caspase activation suggests that a recovery period of at least 3h at 37°C is necessary to visualize caspase activation [44]. Our preliminary experiments analyzing cleaved caspase-3 suggest that a recovery period of 5-6h at 37°C is optimal in our experimental system (data not shown). Cells were harvested to obtain total protein at the experimental endpoint.

### MTT cell viability assay

Cells were first plated at 10,000 cells per well in 24-well plates and subsequently treated with mild hyperthermia or heat shock, as per the experimental requirements. MTT reagent [3-(4,5-dimethylthiazol-2-yl)-2,5-diphenyltetrazoliumbromide] (Sigma-Aldrich, St. Louis, MO) was added at 1X concentration. Following 4h incubation at 37°C, the media containing MTT reagent was replaced with isopropanol (600uL/well). Following 20 min incubation at 37°C to dissolve the formazan crystals, 200uL of final reaction product was transferred to different wells of a 96 well plate and the absorbance was measured at 570nm.

### Protein isolation

Total protein isolation was performed as previously described [25]. Briefly, cell pellets were lysed with RIPA lysis buffer (Sigma, St Louis, MO) supplemented with a protease inhibitor cocktail (Sigma), HALT phosphatase inhibitor (Pierce, Rockford, IL), and 1mM DTT. Protein lysates were incubated for 30 min on ice and subsequently centrifuged at 10,000Xg. The supernatants were collected and stored at −80°C.

### Western blotting

Western blotting was performed as previously described [25]. Briefly, aliquots containing 40μg (for dicer western blots from cell lysates derived from HeLa cells), 20μg (for dicer western blots from cell lysates derived from MEF cells, and 20μg (all other proteins) of total protein were prepared. Samples were then supplemented with SDS buffer and 0.13M DTT (Sigma) and run on 7% (for dicer) or 10% (for all other proteins) SDS polyacrylamide gels. The kaleidoscope molecular weight ladder (250 – 25kDa) from BioRad was used in all western blots. Samples were then transferred to PVDF membranes (BioRad) and blocked with 5% milk in TTBS (Tween 20 Tris-Buffered Saline). Samples were blocked with 5% BSA in TTBS for the analysis of phosphorylated proteins. Subsequently, PVDF membranes were probed with the appropriate primary and secondary antibodies, developed with West Pico Chemiluminescent substrate (Pierce), and analyzed on X-ray film (Thermo Scientific, Waltham, MA). For experiments involving the simultaneous analysis of multiple proteins, PVDF membranes were cut to include sections containing proteins of the appropriate molecular weight prior to antibody incubation. For experiments involving the detection of phosphorylated proteins, the phosphorylated form of the protein was first detected by the method described above. Subsequently, the PVDF membrane was stripped with 0.1N sodium hydroxide, neutralized and re-probed with the appropriate primary antibody to detect all forms of the same protein. Western blots were quantified by scanning X-ray films. Protein band intensities were analyzed using the Image J image processing and analysis tool available at http://rsbweb.nih.gov/ij/. GAPDH served as the loading control for all proteins analyzed, unless indicated. Band intensities were normalized to the loading control within each biological replicate. The representative GAPDH gel images shown in each figure corresponds to the dicer gel image in the figure.

### Dicer activity assay

A pre-miR-124a template was first generated via PCR using HeLa cell genomic DNA. The primers used to generate this template were designed using the miR-124a sequence made publically available through miRBase (mirbase.org). A T7 recognition element was included in one of the primers to facilitate *in vitro* transcription in a subsequent step. Pre-miR-124a was chosen for this assay because it is not endogenously produced in the cell types used in this study. The pre-miR-124a template was transcribed *in vitro* using the MegaScript T7 *in vitro* transcription kit (Ambion, Foster City, CA), together with α^32^P-UTP (Perkin Elmer, Waltham, MA) to generate a α^32^P-UTP radiolabeled pre-miR-124a. This radiolabeled pre-miR-124a was used in all of the activity assays. Protein lysates from HeLa cells grown at 37°C or at 39.5°C, and protein lysates from wildtype and dicer LOF MEFs at 37°C were subsequently incubated for 16 hours with the radiolabeled pre-miR-124a. Once the reactions were complete, the samples were run on a denaturing Urea PAGE gel and specific mature-miR-124a cleavage products were quantified by exposure to a phosphorimaging cassette (Molecular Dynamics Storm).

### siRNA transfection

Control and dicer siRNAs targeting DICER1 in Homo sapiens and Mus musculus were purchased from Qiagen (Valencia, CA). SiRNA transfections were performed in accordance with the Jet Prime siRNA and plasmid DNA transfection kit protocol (VWR International, Radnor, PA). Transfections were carried out at 80nM for both dicer and control siRNAs. Total protein was isolated at 48h and 72h post-transfection for the analysis of dicer knockdown via western blotting.

### Antibodies

Primary antibodies: Dicer (human), ATF4, CHOP, Caspase-3, total and phosphorylated eIF2α (Ser51), total and phosphorylated PKR (Thr451) (Cell Signaling Technology, Boston, MA); Dicer (mouse) (Bethyl laboratories, Montgomery, TX); GAPDH, HSP90, HSP70 (Abcam, Cambridge, MA). Secondary antibodies: Anti-mouse IgG HRP conjugate and anti-rabbit IgG HRP conjugates (Promega, Madison, WI).

### Reagents

PKR inhibitor [8-imidazol-4-ylmethylene-6H-azolidino 5,4-g benzothiazol-7-one (Calbiochem-EMD Millipore, Billerica, MA)] was used at a concentration of 250nM for validation experiments and with hyperthermia treatments. RNase inhibitor (Protector RNase inhibitor, Roche Life Sciences) was used (1.0 unit per reaction) in dicer activity assays.

### Statistical analysis

The Student's t test function in SPSS 22.0.0.0 was used to determine statistical significance and p< 0.05 was considered statistically significant. Values shown are mean ± SEM.

## Abbreviations

ATF: activating transcription factor
ATF6f: activating transcription factor 6 (cleaved)
ASK1: apoptosis signal-regulated kinase 1
BIP: binding immunoglobulin protein
BCL-2: B-cell lymphoma 2
BCL-XL: B-cell lymphoma XL
CHOP: C/EBP homologous protein
CYPB: cyclophilin B
ER: endoplasmic reticulum
ERAD: ER associated degradation
eIF2α: eukaryotic initiation factor 2 alpha subunit
ERP72: ER protein 72kDa
GAPDH: glyceraldehyde 3-phosphate dehydrogenase
GCN2: general control non-derepressible 2
GRP78: glucose regulated protein 78kDa
HSF1: heat shock factor 1
HSP70: heat shock protein 70kDa
HSP90: heat shock protein 90kDa
HRI: heme regulated interferon
IRE1α: inositol requiring enzyme 1α
JNK: jun N-terminal kinase
miR: microRNA
MEF: murine embryonic fibroblast
PERK: PKR-like endoplasmic reticulum kinase
PKR: protein kinase R
TNF: tumor necrosis factor
XBP1: X-box binding protein 1
XBP1s: X-box binding protein 1s.
